# Sustainability and scalability of egg consumption in Burkina Faso for infant and young child feeding

**DOI:** 10.3389/fnut.2022.1096256

**Published:** 2023-01-11

**Authors:** Emily V. Moore, Elizabeth Wood, Heather Stark, Aissata Wereme N'Diaye, Sarah L. McKune

**Affiliations:** ^1^Department of Environmental and Global Health, University of Florida, Gainesville, FL, United States; ^2^Center for African Studies, University of Florida, Gainesville, FL, United States; ^3^Kamboinsé Agricultural Environnemental Research and Training Center, Institut de l'Environnement et de Recherches Agricoles, Ouagadougou, Burkina Faso

**Keywords:** nutrition, sustainability, animal source foods, child health, behavior change

## Abstract

**Introduction:**

Malnutrition is a significant challenge to the health of women and children in Burkina Faso. Given the critical role of animal source food on the health of infants and young children (IYC), interventions continue to explore the potential for eggs to prevent malnutrition.

**Methods:**

Using data from the Un Oeuf intervention, which significantly increased IYC egg consumption, combined with quantitative and qualitative data from endline and 3-month follow-up, we explore the barriers and facilitating factors to IYC egg consumption and the sustainability and scalability of the intervention.

**Results:**

Child egg consumption was high at follow-up in the Control, Partial, and Full Intervention arms (83.3, 88.2%, and 100, respectively). The Full Intervention arm had the highest mean number of eggs consumed (2.9, 2.6, and 5.7), which reflected a slight reduction from endline (6.2). All participants owned chickens at follow-up (100%), however, flock size varied. The Full Intervention arm had more chickens (mean 8.8) than the Control (5.1) or Partial Intervention (6.2) arms, which was a 50% reduction in below endline (18.5 chickens). Qualitative results indicate that chicken ownership, education about the nutritional value of eggs, and spousal support facilitated IYC egg consumption. Barriers included egg production, cultural taboos, and animal health. Motivational factors reported included the observed improvement in child health, increased availability of mothers' time, and mothers' financial independence. Knowledge sharing within the Full and Partial Intervention groups was widely reported, and the sustainability of IYC egg consumption was reinforced by accountability among mothers and to community leaders, flipbooks distributed during the project, and high motivation.

**Discussion:**

Main findings indicate that mothers who received the full Un Oeuf intervention were able to overcome barriers to feeding their child an egg daily, were able to improve their livelihood, were motivated to continue feeding their child eggs, and saw the addition of eggs into the child's diet as sustainable. Future nutrition sensitive agriculture interventions should consider tailoring this approach for other LMIC contexts. Future research is needed to explore a possible threshold in the number of household chickens necessary to continuously feed a child an egg a day.

## Introduction

Animal source foods (ASF) have been identified by the World Health Organization (WHO) as the best high-quality, nutrient-rich food for children aged between 6 and 23 months (WHO, 2014). Due to their critical importance in the growth and development of infants and young children, several studies have sought to examine the impact of ASF consumption on child growth and development in low- and middle-income countries (LMIC) (1–4). Results in meta-analysis are varied and inconclusive in understanding the full impact of ASF on child growth and development, with authors indicating that the variability in study design and inconsistency in definitions of exposure (ASF consumption type, quantity, etc.) and outcomes (stunting, wasting, underweight, etc.) inhibit quantitative analysis across studies (5). The nutritional value of ASF, specifically eggs, is distinctly suited to support early child growth and development (6–8); thus, research continues to explore the use of ASF to improve the growth and development of children during key windows of development (9), particularly those in LMIC. One such study, the *Un Oeuf* study, was a cluster randomized controlled trial conducted in Burkina Faso (10). The *Un Oeuf* study tested a behavior change strategy geared toward increasing egg consumption in infants and young children (IYC) and found significant increases in both egg consumption and improvements in child growth outcomes (11). Given the near-ubiquitous nature of poultry in small-scale farming households within LMIC, this work aims to understand both the sustainability and scalability of the behavior change intervention implemented through the *Un Oeuf* study. Additionally, a follow up study was conducted 3 months after the completion of the *Un Oeuf* study to assess facilitating factors and child egg consumption. This paper examines follow-up data from the *Un Oeuf* endline and follow- up study, to better understand the motivation and experience of the participants in the original *Un Oeuf* study and examine the sustainability and scalability of the behavior change intervention to increase ASF consumption in IYC.

## Background

Burkina Faso is a low-income country in the Sahel region of Sub-Saharan Africa (SSA) with numerous poor development indicators, including high mortality rates among children under five (CU5), neonates, and infants (12). A significant challenge to child health in Burkina Faso is malnutrition—with high rates of malnutrition (8.1% wasting), anemia (77%), and stunting (26.1%) in CU5 (13, 14). Much of this is attributable to high levels of food insecurity, inadequate complementary feeding practices, poor dietary diversity, and a general lack of food availability (15).

In Burkina Faso, ASF consumption is low particularly among women and children (16, 17). Barriers to ASF consumption, such as cultural beliefs and stigma surrounding egg consumption by children, may significantly constrain the consumption of ASF and in particular chicken eggs in Burkina Faso (1, 18). Many studies have shown the importance of including livestock derived ASF (milk, meat, dairy, and eggs) in a child's diet, especially during the critical window of child development from conception through 2 years old (9, 19–21). Regular consumption of ASF has been shown to improve the growth, nutritional status, cognitive development, and overall health of a child (22). Additionally, there is evidence showing the essential role mothers play in improving childhood nutrition (23, 24), and there is growing evidence that targeting and empowering female caregivers of children through livestock production and programming may improve child nutrition through increased ASF consumption (11, 25).

Within Burkina Faso, livestock is typically produced for income, gifting, and socio-religious practices, as opposed to production for direct, household consumption among household members (10). Consequently, innovative approaches that encourage, facilitate, and ultimately increase ASF consumption among rural livestock holders are needed. Using successful seminal egg studies from Ecuador (1) and Ethiopia (26) as guides, the *Un Oeuf* study aimed to increase ASF consumption in IYC through an innovative intervention that involved the gifting of chickens by a community champion and a culturally tailored behavior change strategy to improve livestock production and empower women (10, 11). Results from the intervention showed a significant increase egg consumption (11). Due to the high level of behavior change observed prior to the end of the intervention, additional funding was sought and secured to add a qualitative research component and a three-month follow-up survey to examine the facilitating factors and barriers to egg consumption within the context of the study. Leveraging these FGD and survey data this study aims to (1) examine and describe the facilitating factors that allowed for behavior change observed in the *Un Oeuf* study, and (2) examine the sustainability and scalability of the behavior change intervention to increase ASF consumption in IYC.

## Materials and methods

### Study setting

This research was conducted in 18 rural villages in the Kaya region of Burkina Faso. Villages were in a resource poor setting and comprised of households whose livelihoods were based on smallholder farming. Households in these villages were dependent on a mix of crop and livestock for production and had very low levels of literacy (14.6%), sanitation (23.8% open defecation), and dietary diversity, and high rates of malnutrition (wasting 10.8%, stunting 21.6%) (10).

### Study design

Given that the study presented here builds on the *Un Oeuf* study, a brief overview of that study is presented here. The *Un Oeuf* study had three research arms—(1) a Full Intervention group, whose child participants were gifted chickens by a community champion at the onset of the project and maternal participants received monthly Integrated Nutrition and Agriculture (INA) trainings for 10 months; (2) a Partial Intervention group, whose participants received the same monthly INA trainings as the Full Intervention group but no chickens until the end of the study, at which time they received two chickens; (3) and a Control group, whose participants received no trainings, but, like the Partial Intervention group, received two chickens upon completion of the study (10). These research arms are preserved for the study presented here. Following the completion of *Un Oeuf* , a closing ceremony recognized the efforts of the participating women and women from the Full Intervention Group educated and trained women in the Control group so that they would benefit from INA training that focused on the importance of feeding your child an egg a day.

This study engages a mixed-methods research approach, analyzing qualitative data alongside existing and new quantitative data. The study examines data from 247 surveys conducted during a follow-up study and analyzes qualitative data collected at endline and follow-up with mothers in 9 of the 18 participating villages (see full *Un Oeuf* sampling strategy and previously published findings (10, 11).

### Quantitative sample, data collection, and analysis

Questions from the *Un Oeuf* study household survey were used for quantitative data collection at follow-up. These included child egg consumption (prevalence and number); poultry production (chicken ownership and number); and mothers' decision-making. To measure egg consumption mothers were asked to report on foods that the child had eaten in the past 7 days and, when eggs were indicated, additional probes quantified the number of eggs. Household poultry ownership was reported as yes/no, and if yes, how many. Participants were asked four questions about household decision-making—*who decides* (1) what foods to feed the children, (2) which foods to purchase, (3) how food is portioned, and (4) what to do with household eggs? Household decision-making variables were coded as binary “self” or “other.” Data were managed and analyzed using IBM Statistical Packaging for the Social Sciences (SPSS), and descriptive statistics from follow-up are presented in Table 1.

**Table 1:** Egg consumption, household chicken ownership, and household decision-making (HHDM) at the three-month follow-up of the *Un Oeuf* study population by research arm.

**Follow-up summary statistics**				
	**Control** ***n*** = **84 (%)**	**Partial** ***n*** = **85 (%)**	**Full** ***n*** = **78 (%)**	**Total** ***n*** = **247 (%)**
Egg consumption^*^	70 (83.3)	75 (88.2)	78 (100)	223 (90)
Mean	2.9	2.6	5.74	3.70 (2.19)
Mode	3	2	7	2
Range	7	6	9	10
**HH chicken ownership** ^**†**^	84 (100)	85 (100)	78 (100)	246 (100)
Mean	5.12	6.18	8.77	6.64 (3.31)
Mode	4	6	6	6
Range	13	11	27	28
**HHDM**				
**Foods for children**				
Self	84 (100)	85 (100)	78 (100)	247 (100)
Other	-	-	-	0 (0)
**Foods purchased**				
Self	-	-	2 (2.6)	2 (0.8)
Other	84 (100)	85 (100)	76 (97.4)	245 (99.2)
**Food portions**				
Self	84 (100)	84 (98.8)	79 (100)	245 (99.2)
Other	-	1 (1.2)	-	2 (0.8)
**Household eggs**				
Self	55 (65.5)	55 (64.7)	51 (65.4)	161 (65.2)
Other	29 (34.5)	30 (35.3)	27 (34.6)	86 (34.8)

^*^The number (percent) of children reported to have eaten eggs in the past week.

^†^The number of households that own one or more chickens, followed by the mean, mode, and range of the number of chickens owned.

### Qualitative sample, data collection, limitations, and analysis

A stratified purposive sampling frame was used to select nine of the 18 villages for qualitative data collection. The average number of eggs consumed by the targeted child at midline of the *Un Oeuf* study was used to stratify villages into three groups—low, medium, and high egg consumption. The village with the lowest, average, and highest egg consumption rates at midline (n=3) in each research arm (Full, Partial, and Control; for a total of nine villages) were selected for participation in qualitative data collection.

Qualitative data were collected through FGDs conducted at two time periods: May 2019, immediately following the endline survey of, and August 2019, after follow-up surveys. The FGDs were facilitated using a set of open-ended questions which aimed to identify and explore facilitating factors, barriers, household dynamics, and community-level perceptions relevant to behavior change. Focus group discussions consisted of seven open-ended questions; the guide is included in Supplementary material to this article. Given the difference in the nature of their experience in the study, language in the open-ended questions varied slightly across research arms. Focus groups were administered using a team of three Burkinabe researchers, two of whom were heavily involved with project implementation and quantitative data collection and a local translator with community rapport. The FGDs were conducted using one researcher to facilitate the FGDs in the native language of Moré, whilst the other two researchers simultaneously took separate notes. Despite training and appropriate efforts by data collectors, transcripts and field notes indicate that women engaged in the FGDs more like a group interview, where not all women engaged equally in discussion. Following the collection of data, all notes were compiled and checked against the audio recording to ensure a complete capture of each discussion. Data were then translated into a master set of English transcripts for all nine villages.

The FGD transcripts were independently manually coded by two researchers at a US-based university, using thematic analysis to capture salient themes (27). Themes were coded based on patterns that manifested deductively and inductively between the two reviewers. Themes that were inductive were informed by USAID's cross-cutting themes that were considered priorities within the scope of this project. In order to ensure trustworthiness of the data, inter-rater reliability and consensus were established by comparing and negotiating themes independently derived between the two researchers (28). The final list of themes and sub-themes is presented in Table 2. No qualitative research software was used for analysis.

**Table 2:** Taxonomy of themes and subthemes from focus group discussions with the *Un Oeuf* study participants by research arm.

**Theme**	**Subtheme**	**Content by research arm**		
		**Control**	**Partial intervention**	**Full intervention**
**Facilitating factors** *A facilitating factor is anything that helped facilitate feeding eggs to the enrolled child*	Household chicken ownership	“[The project] increasing the hens.” “We received chickens and now we are able to feed our children eggs.”	“[U]sing our hens' eggs for our children. What would have helped us [more] is the donation of hens at the beginning of training.” “The increase [to] our bird stock [from the project].” “We [bought] hens for our children.	“Chicken donations help women to feed their children eggs.”
	Education	“…[A]sking questions each month changed our behavior toward feeding our child eggs (or other foods).”	“The messages from the trainings that will stay with us are: a child an egg daily; handwashing prevents the spread of diseases.”	“…[A] child an egg daily, we must clean very well our house and henhouse.” “We learned how to take care of our hens, household, and child.”
**Facilitating factors** *A facilitating factor is anything that helped facilitate feeding eggs to the enrolled child*	Spousal Support	“[The spouses support us] by giving us permission to participate in the survey.” “My husband built a hen house for the hens [donated by the project] and paid [for] a rooster to add to the hens…”	“Our spouse's hen donation [help feed our children].” “My husband has built a chicken house for my chickens.” “My husband often calls the vet to vaccinate our chickens.”	“[Our husbands] encourage us to participate in the training.” “Our husbands often help us in breeding chickens.”
**Barriers** *A barrier is anything that prevents the feeding of eggs to the enrolled child*	Egg production	“The low number of [egg] laying hens.” “If our hens don't eat well, they cannot lay eggs.”	“When hens brood and we do not have money to buy eggs.” “We [do] not have many hens.” “The [two] hens do not lay enough eggs.”	“If the hens are sick and do not lay.” “If the hens are not well fed, they will not lay [eggs].” “…[A]t the beginning the hens were [too] young.”
	Cultural taboos^a^	“Barriers keep us from feeding our child an egg daily, the social and cultural barrier [that] a child must not eat eggs.”	“At the beginning we did not feed children eggs because of traditional barriers, but since we received the training, we are feeding our children eggs.”	
**Barriers** *A barrier is anything that prevents the feeding of eggs to the enrolled child*	Animal health	“When hens do not have a hen house; they lay eggs where they want.” “[H]ens lack food.” “The non-existence of a hen house for hens…if it rains, I am obliged to put my hens and their chicks in our house…” “Avian pathologies can decimate hens.” “[M]aintenance and follow-up of the hens.”	“Unfortunately, I only have one hen now, the other hens are dead, so I can't get eggs for the child. I have to buy eggs for my child.” “When there is no local veterinarian to vaccinate our hens… they will become sick.”	“If we don't have medicines to give to the hens when they are sick.” “If we don't have hen houses.” “[I]f you do not have hen houses, it will be difficult to take care of poultry.” “How are we going to take care of our poultry without you?”
**Motivational factors** *A motivational factor is anything that motivated and inspired the mothers to start or continue feeding eggs to the enrolled child*	Health of child	“Our child is very healthy, compared to other children, his weight is normal.”^b^ “…[C]hicken eggs improve his growth and intelligence.”	“All my children were malnourished and since I started giving eggs to this child, he is doing well. [He] is not malnourished [like] his other siblings.”	“[I] can see an impact of the project on our child. [He is] very healthy, are in top form. [He is] are growing well compared to other children his age who are not enrolled.”
**Motivational factors** *A motivational factor is anything that motivated and inspired the mothers to start or continue feeding eggs to the enrolled child*	Health of child	“…[E]ating eggs helps children to avoid some diseases.	“The fact that we see our children are healthy motivates [us].”	“…We have more respect for our community leader, to know they turned [our] attention to our children's nutrition shows we must take care very well of children's hens.”
	Time	“There is the decrease of breastfeeding of children thanks to the eggs.” “There is Partial release of mothers and increase mothers' household time.” “There is the saving of time (and money) by mothers thanks to the good health of the children.”	“There was a change, the children suckle less and are healthy. [W]e are also healthy.”	“As a mother, [I am] satisfied there is a decrease in child breastfeeding through egg consumption.” “When a child eats an egg, he suckles less and gives a lot of free time to the mother to do her activities.”
**Motivational factors** *A motivational factor is anything that motivated and inspired the mothers to start or continue feeding eggs to the enrolled child*	Financial independence	“We will have profits because we [now] have chicks [from the project donation] and they will become chickens we will sell some of them to support certain needs.”	“As mothers, we are happy. It will benefit [us] if we have many chickens; we can sometimes sell some chickens to support our child's needs or our needs.”	“I am happy to have chickens. I can often sell some chickens to pay [for] my child's clothes.” “We learned a lot about poultry breeding … now we can take care [of] or breed poultry ourselves.” “I sold some chickens to pay for a small ruminant for my child.” “As a mother, we see a difference [between] other mothers who did not receive the chickens. The mother who received the chickens is financially independent…”
**Knowledge-sharing** *Mothers within the full and Partial Intervention arms were sharing their knowledge, whilst mothers in the Control were eagerly accepting of it when shared*	Community	“We heard about the program from one of our family members.” “…[T]he project has changed how we interact within our household and our community, because we tell other women in our community what we learned about children's nutrition during the survey.” “…[O]ther women in the village put into practice the advice and some would like to participate in the program.”	“[We shared] the benefits of egg consumption, child hygiene, and sanitation.” “When we go home after training sessions, we share what we learned with our neighbors.” “[We shared this information] because it will help other women to take care of their children.”	“…[W]e use the flipbooks to share information with women outside our community.” “We share this information with women outside in our community (village) who are participating in this project.” “[This] benefited us, so we want the same thing for [other mothers]”.
	Household	“I shared this with my husband's second wife.” “In our household, the project has changed our behaviors around health and hygiene of [our] children.”	“There is the involvement of household members in poultry monitoring.” [We share information] with our husband's second wife.”	“Behaviors [that] have changed in our household are our children's hygiene and nutrition, [and] poultry's hygiene.”
**Sustainability** *Sustainability within the population is what the participants planned to do to maintain egg consumption within their households*	Behavior Reinforcement	“We will take care of the chickens to always have eggs.” “We will remind each other what we must do.” “We will take care of the chickens to have eggs at all times.”	“We will use our flipbooks. Our flipbooks contain information that helps us to put into practice what we learned during training sessions.” “We have our flipbooks that will help us to continue [to] remember everything we learned during our training and put it into practice	“We will use our flipbooks to remember.” “We will vaccinate our chickens. [T]here are people in the villages who can vaccinate our chickens.” “[I] will always apply the creed: ‘a child an egg a day,' even if [I] give birth to another child.”

^a^There were no reports of cultural taboos being a barrier within the Full Research Arm.

^b^It is important to note that mothers in the Control Group mentioned the health of their children in relativity to no other children that were enrolled in the study. This shows the skewed perspective of health that can occur when a village has low dietary diversity and faces food insecurity.

## Results

The results presented in this analysis consist of quantitative and qualitative data collected at the end of the study (FGD) and at a 3-month follow-up (FGD and follow-up surveys). Quantitative research-arm-level findings from baseline and endline of the *Un Oeuf* study have been included in Supplemental material to facilitate comparison with the follow-up data presented below.

### Household survey

At 3-month follow-up, following the end of the *Un Oeuf* study, 247 participants mothers were surveyed. The results for egg consumption, household chicken ownership, and household decision-making can be found in Table 1. Follow-up survey data indicate that egg consumption expanded through follow-up; 223 children (90%) consumed eggs the week prior to data collection, with an average consumption of 3.7 eggs per week. In the Full Intervention group, all children (100%) were consuming eggs at follow-up, compared to 83.3 and 88.2% in the Control and Partial Intervention groups, respectively. In all three research arms, all households reported owning chickens (100%) at follow-up, with an average number of chickens of 6.64 (range 2–30). The average number of chickens remained higher in the Full Intervention group than the Partial Intervention group (8.8 compared to 6.2), both of which were higher than the Control group (5.1). Very little difference was observed in decision-making at follow-up: 100% of all mothers reported making household decisions about what foods are fed to children; and 99% (all but one mother) reported being the decision maker about how foods are portioned. Two women (0.8%) reported making decisions about what foods to purchase; importantly, both women were in the Full Intervention arm. Finally, 161 women (65%) in the study population reported being decision maker about what is done with the household eggs; distribution was comparable across all three groups (65.4, 65.5, and 64.7% in the Full, Control, and Partial Intervention groups, respectively).

### Focus group discussion

The FGDs yielded six prominent themes—facilitating factors, barriers, motivational factors, livelihood, knowledge-sharing, and sustainability (Table 2). Each theme has been operationalized with a definition provided in Table 2.

### Facilitating factors

Within the theme of facilitating factors, three subthemes were identified—household chicken ownership, education, and spousal support. At follow-up, all research arms reported that an increase in the household chicken ownership facilitated the mothers to feed the enrolled child eggs. While the delivery of education varied across research arms, there was consensus that increasing knowledge (and awareness) was a key facilitating factor in the behavior change of feeding eggs to children—including the Control group which only received educational materials in the form of a “flipbook” when they received chickens for the household at the end of project ceremony. Unintended education for the Control group came in the form of the household survey, which brought attention to and started conversations on feeding eggs (and other nutritionally rich foods) to children. For the Partial and Full groups, the education was much more formalized through the implementation of INA trainings that were attended each month, as well as educational materials (i.e., flipbooks) that were given to mothers. That mothers kept and owned their own flipbook, which facilitated their ability to refer to the flipbooks at any time, was a key component in the education on behavior change toward feeding children eggs.

There was a consensus in all villages that the support of husbands was integral to facilitation of egg consumption, including the relinquishing of household decision-making over what the child(ren) consumed. Women reported that their husbands were supportive and encouraging of the women's involvement in the study, and that they helped facilitate egg consumption by giving eggs or hens from the household flocks, purchasing chickens so eggs would be available, building hen houses, and helping in the care of the chickens.

### Barriers

The theme of barriers consisted of the subthemes of egg production, cultural taboos, and animal health. Importantly, women in the Full Intervention arm reported that any barrier present and initially limiting egg consumption were overcome by the study design.

The lack of egg production was a barrier for all research arms. As expected, women reported that hens do not lay eggs when sick. Additionally, at the beginning of the intervention, hens in the Full Intervention arm were too young to lay eggs; therefore, women in the full group experienced a lack of egg production due to having young hen flocks. Women reported that when hens brood or laid no eggs, it was a burden to need to purchase eggs for the child.

Within this region of Burkina Faso, a cultural taboo surrounding the consumption of eggs by children—particularly young girls—was identified as a barrier to egg consumption by participants. This taboo was identified during formative research and therefor included and addressed in early training sessions of the mothers. With support of community champions, acting as advocates for egg consumption, once women understood that consuming eggs was beneficial to a child's health, this taboo no longer limited women's willingness to feed children eggs in the Partial and Control research arms.

Another consistent barrier across research arms was the health of the animals. Women expressed that when they lack the ability to properly feed their hens, the hens fall ill, with consequences on egg production and on flock size. Additionally, the financial barriers to construct a hen house, which also then implied feeding the hens, left the birds subject to predators and weather. Limited vaccine availability was also identified as a cause for poor hen health.

### Motivational factors

Child health and time-gain of the mothers were the two subthemes deduced from the theme of motivational factors. The health of the child was the overarching and most-reported motivational factor across all groups. Mothers all agreed that the most motivating reason behind their behavior change of feeding the children eggs was the improvement in the children's nutrition, growth, and overall health.

Mothers agreed that with the addition of eggs in the children's diets, the children demanded to suckle less; therefore, reducing the time-demand on the mother. Because of this release from breastfeeding, mothers reported this increase in available time as a motivational factor because they were able to better care for themselves and their households.

### Livelihood

Within livelihood, the subtheme of financial independence arose, strictly surrounding the benefits yielded from poultry farming. Mothers agreed that there was a newfound sense of financial independence due to the increase that poultry production brought them. Particularly in the full group, women reported being able to purchase clothing for her children, pay for school fees, as well as purchase small ruminants and other foods to increase the dietary diversity of the children.

### Knowledge-sharing

Knowledge-sharing was shown within two subthemes—community and household. Knowledge-sharing within the community, both at the village-level and broader department-level, was witnessed across all research arms. Once mothers had knowledge to share, they shared it. Women explained they did this so that other women would have healthier children. This knowledge was shared for the good of the greater community. Knowledge-sharing within the households took place between the mothers who were enrolled in the study and their husband and co-wives. This knowledge was exchanged for the betterment of the entire family unit.

### Sustainability

Sustainability in the sense of behavior reinforcement was expressed across all three research arms. Since behavior change is an iterative process, behavior reinforcement is key in sustaining behavior change. Women explained their desire to always properly care for their chickens to ensure their children always had eggs. Additionally, women stated that they would continue to use and share the information in the flipbooks, provided by the project. The flipbooks contained picture-based information about poultry management, child diet, and IYC feeding practices, all elements that the women saw as reminders of what is needed to improve child health through poultry production. Women appreciated, shared, and valued the flipbooks.

## Discussion

This study was launched in order to identify factors that allowed for significant increase in IYC egg consumption during the *Un Oeuf* study and to assess the sustainability and scalability of the approach. As previously published, at endline of the intervention, 100% of children in the Full Intervention arm were consuming eggs (11). The results of the qualitative data revealed that women in the Full Intervention group were able to overcome any barriers that presented at the beginning of the study, with attribution to intervention design elements, including training (increased education and understanding of eggs' nutritional value) and livestock assets (increased flock size), both embedded in a culturally sensitive approach (the support and advise of a trusted community champion during the gifting ceremony). Important motivational factors identified by the women included the observation of improvements in child health and increased time availability. Observation of visible improvements in child health acted as a natural reinforcer for mothers feeding their children eggs. Natural reinforcers, a type of positive reinforcement (improved health) that occurs because of a behavior change (egg consumption), are well-documented in behavioral psychology as instrumental to sustained behavior change (29, 30). No previous studies were identified that documented mothers' observation of IYC growth as motivational for adherence a nutritional intervention in a LMIC. The alleviation of time poverty that afflicts mothers (31, 32), allowed the mothers to better care for all members of their households, including themselves, a finding that supports previous research (33, 34). Mothers also reported new levels of financial independence, a critical pathway for improving maternal and child health and nutrition (25, 35, 36). While facilitating factors such as education about the nutritional value of eggs and ongoing spousal support allowed for continued IYC egg consumption at follow-up, egg availability emerged as an important constraint, limiting the frequency of IYC egg consumption, even among women where motivation remained high.

Data presented here indicate that the high prevalence of IYC egg consumption in the Full Intervention group persisted at 3-month follow up (100%). However, the number of eggs consumed per week dropped from 6.3 to 5.7 from endline to follow-up. This drop was matched by a precipitous drop in chicken ownership: while all households in the Full Intervention arm had chickens at endline and at follow-up (no change, 100% at each observation), the mean number of chickens dropped from 19 chickens to 9 by follow-up. These data reflect that which was described in FGD, that women in the Full Intervention group were highly motivated at endline and at a 3-month follow-up to feed their child an egg a day, but they began to face some constraints related to poultry numbers by follow-up.

Across research arms, mothers reported the number of chickens and associated number of eggs to be one of the most important determinants to their ability to feed their child an egg. This supports findings from previous research, which finds important associations between animal ownership and ASF consumption, most often milk, poultry, and eggs (25, 37–40). Women in the Full Intervention group credited the influx of (four) chickens into their household as being life changing for their livelihoods, while mothers in the Partial Intervention and Control groups stated that the project's donation of two hens at endline was instrumental in creating behavior change, as well as freeing up household income previously spent on purchase of eggs. Focus group discussions indicated that animal health, including vaccination, housing, and other key management strategies, were instrumental in increasing poultry production (number of chickens) and productivity (number of eggs). The INA trainings that addressed these issues were highly valued by participants seen as contributing to the increased flock numbers and egg consumption at endline. This was supported by some literature (41), but other interventions that have taken similar tacks did not succeed in increasing poultry production (42). Limitations, such as the inability of the project to assist women in the *Un Oeuf* study with construction of chicken houses, were also identified.

Despite the prevalence of child egg consumption increasing between endline and follow-up in the Partial Intervention and Control arms to percentages much closer to the Full Intervention arm, the mean number of eggs consumed per week by children in the Partial Intervention and Control groups remained lower (2.6 and 2.9, respectively) than in the Full Intervention group (5.7). In conjunction with qualitative findings and the observed drop in poultry numbers and egg consumption in the Full Intervention group, this observation calls into question a possible threshold of poultry numbers sufficient for egg consumption, as the Full Intervention arm had almost nine chickens at follow-up, which was higher than the Partial (6) or Control (5) group. This supports previous research which found the type and number of livestock to be important determinants of any improvements in child diet or growth (37). In addition, the excess poultry production in the Full Intervention group at endline allowed mothers a sustainable means of livelihood – something that could be sold to support the purchase of other household needs, including medicines, other types of foods that increased the household dietary diversity, clothing, and school fees for children. This reinforces livelihood support interventions that focus on utility and economic improvement to address health-related issues (35, 43).

The second objective of the study was to assess the sustainability and scalability of the intervention. As indicated above, the motivation to feed children an egg a day and a relatively strong ability to do so remained high in the Full Intervention group 3 months after the project ended, indicating that the original intervention was sustainable, at least in the short term. The question of scalability—can this intervention be taken elsewhere and replicated—appears to hold strong potential. The concept of gifting chickens to children by a community champion utilized in this project was piloted by Omer et al. in Ethiopia, where comparably strong results were found (26). In the *Un Oeuf* study, the behavior change strategy leveraged this culturally sensitive approach and focused on increasing egg availability, through poultry ownership and education about poultry production, and increasing motivation and decision-making to feed the child eggs, through education about child nutrition. A policy roundtable, held in January 2020 following the end of the project, reinforced the potential of chicken-gifting by community champions (44). Using the socio-ecological model (SEM) the study design actively engaged multiple levels of support, including household (spousal support) and community level (community champions), which proved instrumental (45, 46). Husbands were invited and encouraged to attend the INA trainings with their enrolled wives, and some contributed chickens, either during the initial ceremony with the village leaders or subsequently on their own. This support from husbands was identified as an important facilitating factor to women continuing to feed children eggs over time. This supports existing literature on the important role of men/fathers in IYC nutrition programs (47–50), as well as literature on the multilevel factors that affect child nutritional practices (51). Though future research is needed to confirm how many chickens are required to produce enough eggs to support a standard of one egg per child each day, the *Un Oeuf* approach appears to be scalable to other smallholder farming communities to improve child diets through increased egg consumption. Such an approach would utilize the SEM to design a culturally tailored intervention in which community champions gift poultry to IYC, and mothers participate in tailored monthly INA trainings (in which fathers are welcome) that reinforce best practices in poultry production and knowledge and practices that support leveraging the nutritional value of eggs for optimal child growth.

## Conclusion

The Full Intervention tested in *Un Oeuf* had a transformative impact on the lives of mothers and IYC by altering the ways in which mothers breed chickens, feed their children, and care for their households. At the onset of the study, chicken eggs were sparsely eaten by children, largely due to poverty, lack of knowledge about nutritional value of eggs, and cultural norms that described eggs as part of a chicken's lifecycle rather than a food source (11). The intervention overcame these barriers and behavior change was sustained 3 months following the study. The *Un Oeuf* study contributes to literature which underscores the potential of using cultural pathways to trigger and sustain nutritional behavior change, even when it challenges cultural norms. Nutrition-sensitive, livestock-based interventions in LMIC may consider tailoring the *Un Oeuf* approach to catalyze and sustain egg consumption in children and other undernourished populations.

## Data availability statement

The datasets presented in this study can be found in online repositories. The names of the repository/repositories and accession number(s) can be found below: https://dataverse.harvard.edu/dataverse/livestock-lab-burkina-egg-consumption.

## Ethics statement

The studies involving human participants were reviewed and approved by University of Florida Institutional Review Board Burkina Faso Ethical Review Board. Written informed consent to participate in this study was provided by the participants' legal guardian/next of kin.

## Author contributions

EM contributed to securing funding, data collection, data management, analysis, and primary writing. EW contributed to analysis and primary writing. HS contributed as co-PI to securing funding, study design, implementation, data management, and primary writing. AW contributed as co-PI to securing funding, study design, and implementation. SM contributed as principal investigator to the project to secure funding, study design, implementation, data management, and primary writing. All authors contributed to the article and approved the submitted version.
