# Supplementary material for: Sustainability and scalability of egg consumption in Burkina Faso for infant and young child feeding
Source: Front Nutr. 2023 Jan 11;9:1096256. doi: 10.3389/fnut.2022.1096256 (PMC9874693; doi:10.3389/fnut.2022.1096256)
Supplement: Supplementary file 1 [file Data_Sheet_1.docx]

FOCUS GROUP DISCUSSION INSTRUMENTS

This appendix contains the Focus Group Discussion Instruments for FGDs held at endline and follow-up.

Endline FGD Instrument

**Control Arm**

1. I’d like to start by understanding your overall sense of how the project went. What was the impact of our asking you questions every month? Why do you think we were doing that?
   - *Did our coming to ask questions monthly cause any problems for you, in the community or your household? Can you describe that experience?*
   - *Was your spouse supportive of your participation? How so/why not?*
2. Do you feed your child eggs?
   - *What facilitating factors lead to feed your child an egg daily?*
   - *What barriers keep you from feeding your child an egg daily?*
   - *Has our asking you questions each month changed your behavior toward feeding your child eggs or other foods?*
3. We have been asking you questions over the past 10 months as part of a study on egg consumption. In other communities nearby, we have been testing an intervention that aims to increase egg consumption among children. We did not run the intervention here. Were you aware of the intervention/program, besides what we have told you about it?
   - *How did you hear about the program? What part?*
   - *Did you hear from others that feeding your child eggs was a good idea?*
   - *Do you think your behavior around egg consumption has changed because of anything you heard from others over the past 10 months?*
4. How does your child compare to other children his/her age in terms of growth, health, and development?
5. What about you? Do you feel the project has changed you?
   - *Has the project changed how you interact within your household? Your community?*
   - *Do you feel the project has empowered you or disempowered you in anyway?*
6. What about your household? How has the project changed your household?
7. Do you have any other comments, questions, or concerns that we should know about?

**Partial Intervention Groups**

1. I’d like to start by understanding your overall sense of how the project went. What worked and what didn’t?
   - *Did it cause any problems in the community or your household? Can you describe that experience?*
   - *Was your spouse supportive of your participation? How so/why not?*
2. As you know, our goal was to improve egg consumption among your children in the study. Did it work? Why or why not?
   - *What helped you provide the egg and day?*
   - *What barriers remained that kept you from feeding your child an egg daily?*
   - *Were you able to overcome any of these barriers? How? What would have helped?*
   - *If/when you fed the child, how did she/he respond to the introducing of an egg into their diet?*
   - *Will you continue to try feed your child an egg a day?*
3. Can you see an impact of the project on your child? How does he or she compare to other children his/her age who are not enrolled?
4. What about you? Do you feel the project has changed you?
   - *Has the project changed how you interact within your household? Your community?*
   - *Do you feel the project has empowered you or disempowered you in anyway?*
5. What about your household? How has the project changed your household?
   - *Feeding practices of other children?*
   - *Food security in general?*
6. I’d like to understand more about how the trainings went. Can you describe what you liked and didn’t like about the monthly INA trainings?
   - *Did you enjoy the trainings? Why/why not?*
   - *Was attending the trainings a burden for you? What were the barrier to attending?*
   - *What messages from the trainings will stay with you?*
   - *What behaviors or norms have changed in your household? Give example?*
   - *Did you find the flipbooks to be helpful?*
   - *Did you share this information with women outside of the women in your community who are participating in this project?*
7. Do you have any other comments, questions, or concerns that we should know about?

**Full Intervention Groups**

1. I’d like to start by understanding your overall sense of how the project went. What worked and what didn’t?
   - *Did it cause any problems in the community or your household? Can you describe that experience?*
   - *Was your spouse supportive of your participation? How so/why not?*
2. As you know, our goal was to improve egg consumption among your children in the study. Did it work? Why or why not?
   - *What helped you provide the egg and day, and what barriers remained?*
   - *How did your child respond to the introducing of an egg into their diet?*
   - *In the design of the project, the children received the chickens directly as gifts. Was this important to you?*
   - *In the design of the project, the child received the chickens from a community leader; did this matter to you? How would your behavior have been different if an NGO had given the chickens?*
   - *Will you continue to feed your child an egg a day?*
3. Can you see an impact of the project on your child? How does he or she compare to other children his/her age who are not enrolled?
4. What about you? Do you feel the project has changed you?
   - *Has the project changed how you interact within your household? Your community?*
   - *Do you feel the project has empowered you or disempowered you in anyway?*
5. What about your household? How has the project changed your household?
   - *Feeding practices of other children?*
   - *Food security in general?*
6. I’d like to understand more about how the trainings went. Can you describe what you liked and didn’t like about the monthly INA trainings?
   - *Did you enjoy the trainings? Why/why not?*
   - *Was attending the trainings a burden for you? What were the barrier to attending?*
   - *What messages from the trainings will stay with you?*
   - *What behaviors or norms have changed in your household? Give example?*
   - *Did you find the flipbooks to be helpful?*
   - *Did you share this information with women outside of the women in your community who are participating in this project?*
7. Do you have any other comments, questions, or concerns that we should know about?

Follow-up FGD Instrument

**Control Group**

*I would like to understand how receiving chickens from the project has affected your household, and how you all think the project can address more women and mothers.*

**Receipt of livestock assets (chickens)**

1. Ownership:
   1. Who owns the chickens received from the project?
   2. Who makes decisions about the chickens and the eggs produced from the chickens given by the project?
2. Consumption:
   1. Does your child enrolled in the project eat chicken eggs after receiving chickens from the project?
      1. Why?
      2. Why not?
   2. Do your other children in the household eat chicken eggs after receiving chickens from the project?
      1. Why?
      2. Why not?
   3. Do your children eat chicken eggs as a result of you attending the project ceremony?
      1. Why?
      2. Why not?
   4. What factors facilitate you feeding your child/children chicken eggs?
   5. What barriers keep you from feeding your child/children chicken eggs?
3. Did receiving chickens from the project affect your household in any way?

**Sustainability of eating chicken eggs**

1. Do you think it is important to feed your children chicken eggs?
2. How often can you afford to feed your children chicken eggs?
3. What would make it possible for you to feed your children chicken eggs every day or multiple times a week?
4. Is it important that husbands and fathers be part of the discussion concerning the importance of feeding children chicken eggs?
   1. Why?
   2. Why not?

**Scalability of eating chicken eggs**

1. Do you think it is important for other Burkinabe women to feed their children chicken eggs?
2. Do you think other Burkinabe women DO feed their children chicken eggs?
   1. Why?
   2. Why not?
3. Do you think other Burkinabe women WOULD feed their children chicken eggs if they knew the benefits of feeding their children chicken eggs?
   1. Why?
   2. Why not?
4. Are there any taboos surrounding chicken egg consumption by children that you are aware of that would prevent other Burkinabe women from feeding their children chicken eggs?
5. Is it important that husbands and fathers know the importance of feeding children chicken eggs?
   1. Why?
   2. Why not?
6. Do you think it is important for other African women to feed their children chicken eggs?
7. Do you think other African women DO feed their children chicken eggs?
8. Do you think other African women WOULD feed their children chicken eggs if they knew the benefits?
9. Did receiving the project findings at the ceremony change make you want to feed your child an egg a day?
   1. Why?
   2. Why not?
10. Did receiving the project findings at the ceremony change your behavior in feeding your children chicken eggs?
11. Do you have any advice to the project on how to encourage or support women to feed their children chicken eggs?

**Partial Intervention Group**

*I would like to understand how receiving chickens from the project has affected your household, and how you all think the project can address more women and mothers.*

**Receipt of livestock assets (chickens)**

1. Ownership:
   1. Who owns the chickens received from the project?
   2. Who makes decisions about the chickens and the eggs produced from the chickens given by the project?
2. Consumption:
   1. Does your child enrolled in the project eat more chicken eggs after receiving chickens from the project?
      1. Why?
      2. Why not?
   2. Do your other children in the household eat chicken eggs after receiving chickens from the project?
      1. Why?
      2. Why not?
   3. Do your children eat chicken eggs as a result of you attending the project ceremony?
      1. Why?
      2. Why not?
   4. What factors facilitate you feeding your child/children chicken eggs?
   5. What barriers keep you from feeding your child/children chicken eggs?
3. Did receiving chickens from the project affect your household in any way?

**Sustainability of eating chicken eggs**

1. Do you think it is important to feed your children chicken eggs?
2. How often can you afford to feed your children chicken eggs?
3. What would make it possible for you to feed your children chicken eggs every day or multiple times a week?
4. Is it important that husbands and fathers be part of the discussion concerning the importance of feeding children chicken eggs?
   1. Why?
   2. Why not?

**Scalability of eating chicken eggs**

1. Do you think it is important for other Burkinabe women to feed their children chicken eggs?
2. Do you think other Burkinabe women DO feed their children chicken eggs?
   1. Why?
   2. Why not?
3. Do you think other Burkinabe women WOULD feed their children chicken eggs if they knew the benefits of feeding their children chicken eggs?
   1. Why?
   2. Why not?
4. Are there any taboos surrounding chicken egg consumption by children that you are aware of that would prevent other Burkinabe women from feeding their children chicken eggs?
5. Is it important that husbands and fathers know the importance of feeding children chicken eggs?
   1. Why?
   2. Why not?
6. Do you think it is important for other African women to feed their children chicken eggs?
7. Do you think other African women DO feed their children chicken eggs?
8. Do you think other African women WOULD feed their children chicken eggs if they knew the benefits?
9. What part(s) of this project do you think are most important to helping and getting women to feed their children chicken eggs? (Trainings? Receiving chickens? Receiving the project findings?)
10. How many trainings did you attend before the importance of feeding your child chicken eggs changed your behavior to feeding your child chicken eggs?
11. Do you have any advice to the project on how to get other women to feed their children chicken eggs?

**Full Intervention Group**

*I would like to understand how you have been maintaining the feeding practice of feeding your child a chicken egg each day.*

**Receipt of livestock assets (chickens)**

1. Ownership:
   1. Who owns the offspring of the chickens received from the project?
   2. Who makes decisions about the offspring of the chickens given by the project?
2. Consumption:
   1. Does your child enrolled in the project eat continue to eat chicken eggs, daily?
      1. Why?
      2. Why not?
   2. Do your other children in the household eat chicken eggs after attending the project ceremony?
      1. Why?
      2. Why not?
   3. What factors facilitate you feeding your child/children chicken eggs?
   4. What barriers keep you from feeding your child/children chicken eggs?
3. Did receiving chickens from the project affect your household in any way?

**Sustainability of eating chicken eggs**

1. Do you think it is important to feed your children chicken eggs?
2. How often can you afford to feed your children chicken eggs?
3. What would make it possible for you to feed your children chicken eggs every day or multiple times a week?
4. Is it important that husbands and fathers be part of the discussion concerning the importance of feeding children chicken eggs?
   1. Why?
   2. Why not?

**Scalability of eating chicken eggs**

1. Do you think it is important for other Burkinabe women to feed their children chicken eggs?
2. Do you think other Burkinabe women DO feed their children chicken eggs?
   1. Why?
   2. Why not?
3. Do you think other Burkinabe women WOULD feed their children chicken eggs if they knew the benefits of feeding their children chicken eggs?
   1. Why?
   2. Why not?
4. Are there any taboos surrounding chicken egg consumption by children that you are aware of that would prevent other Burkinabe women from feeding their children chicken eggs?
5. Is it important that husbands and fathers know the importance of feeding children chicken eggs?
   1. Why?
   2. Why not?
6. Do you think it is important for other African women to feed their children chicken eggs?
7. Do you think other African women DO feed their children chicken eggs?
8. Do you think other African women WOULD feed their children chicken eggs if they knew the benefits?
9. What part(s) of this project do you think are most important to helping and getting women to feed their children chicken eggs? (Trainings? Receiving chickens? Receiving the project findings?)
10. How many trainings did you attend before the importance of feeding your child chicken eggs changed your behavior to feeding your child chicken eggs?
11. Do you have any advice to the project on how to get other women to feed their children chicken eggs?
