# Supplementary material for: Sustainability and scalability of egg consumption in Burkina Faso for infant and young child feeding
Source: Front Nutr. 2023 Jan 11;9:1096256. doi: 10.3389/fnut.2022.1096256 (PMC9874693; doi:10.3389/fnut.2022.1096256)
Supplement: Supplementary file 2 [file Table_1.docx]

| **Control Group Summary Statistics** | |  |  |
| --- | --- | --- | --- |
|  | **Baseline** | **Endline** | **Follow-up** |
|  | n=88 | n=87 | n=84 |
| **Egg consumption*** | 7.5 | 25.3 | 83.3 |
| Mean | 0.19 | 0.66 | 2.9 |
| Mode | 0 | 0 | 3 |
| Range | 5 | 5 | 7 |
| **HH chicken ownership†** | 79.5 | 98.9 | 100 |
| Mean | 5.45 | 6.16 | 5.12 |
| Mode | 0 | 2 | 4 |
| Range | 60 | 30 | 13 |
| **HHDM‡** |  |  |  |
| **Foods for children** |  |  |  |
| Self | 74 | 100 | 100 |
| Other | 26 | 0 | 0 |
| **Foods purchased** |  |  |  |
| Self | 8 | 97 | 0 |
| Other | 92.9 | 2 | 100 |
| **Food portions** |  |  |  |
| Self | 54 | 92 | 100 |
| Other | 46 | 8 | 0 |
| **Household eggs** |  |  |  |
| Self | 31 | 30 | 65.5 |
| Other | 69 | 70 | 34.5 |
|  |  |  |  |
|  |  |  |  |
| **Partial Intervention Group Summary Statistics** | | |  |
|  | **Baseline** | **Endline** | **Follow-up** |
|  | n=89 | n=86 | n=85 |
| **Egg consumption*** | 1.1 | 77.9 | 88.2 |
| Mean | 0.02 | 2.35 | 2.6 |
| Mode | 0 | 2 | 2 |
| Range | 2 | 7 | 6 |
| **HH chicken ownership†** | 77.5 | 97.7 | 100 |
| Mean | 8.53 | 9.4 | 6.18 |
| Mode | 0 | 10 | 6 |
| Range | 100 | 40 | 11 |
| **HHDM‡** |  |  |  |
| **Foods for children** |  |  |  |
| Self | 67 | 97.6 | 100 |
| Other | 33 | 2.4 | 0 |
| **Foods purchased** |  |  |  |
| Self | 5.7 | 1.2 | 0 |
| Other | 94.3 | 98.8 | 100 |
| **Food portions** |  |  |  |
| Self | 56.3 | 87.1 | 98.8 |
| Other | 43.7 | 12.9 | 1.2 |
| **Household eggs** |  |  |  |
| Self | 48.2 | 30 | 64.7 |
| Other | 41.8 | 70 | 35.3 |
|  |  |  |  |
|  |  |  |  |
|  |  |  |  |
| **Full Intervention Group Summary Statistics** | | |  |
|  | **Baseline** | **Endline** | **Follow-up** |
|  | n=83 | n=79 | n=78 |
| **Egg consumption*** | 4.8 | 100 | 100 |
| Mean | 0.14 | 6.25 | 5.75 |
| Mode | 0 | 7 | 7 |
| Range | 7 | 4 | 9 |
| **HH chicken ownership†** | 89.2 | 100 | 100 |
| Mean | 8.63 | 18.51 | 8.77 |
| Mode | 4 | 20 | 6 |
| Range | 100 | 36 | 27 |
| **HHDM‡** |  |  |  |
| **Foods for children** |  |  |  |
| Self | 59.8 | 100 | 100 |
| Other | 40.2 | 0 | 0 |
| **Foods purchased** |  |  |  |
| Self | 7.3 | 3.8 | 2.6 |
| Other | 92.7 | 98.6 | 97.4 |
| **Food portions** |  |  |  |
| Self | 43.9 | 91.1 | 100 |
| Other | 56.1 | 8.9 | 0 |
| **Household eggs** |  |  |  |
| Self | 29.3 | 63.3 | 65.4 |
| Other | 70.7 | 36.7 | 34.6 |
